# Transferability of PCR-based diagnostic protocols: An international collaborative case study assessing protocols targeting the quarantine pine pathogen *Fusarium circinatum*

**DOI:** 10.1038/s41598-019-44672-8

**Published:** 2019-06-03

**Authors:** Renaud Ioos, Francesco Aloi, Barbara Piškur, Cécile Guinet, Martin Mullett, Mónica Berbegal, Helena Bragança, Santa Olga Cacciola, Funda Oskay, Carolina Cornejo, Kalev Adamson, Clovis Douanla-Meli, Audrius Kačergius, Pablo Martínez-Álvarez, Justyna Anna Nowakowska, Nicola Luchi, Anna Maria Vettraino, Rodrigo Ahumada, Matias Pasquali, Gerda Fourie, Loukas Kanetis, Artur Alves, Luisa Ghelardini, Miloň Dvořák, Antonio Sanz-Ros, Julio J. Diez, Jeyaseelan Baskarathevan, Jaime Aguayo

**Affiliations:** 1 ANSES Laboratoire de la Santé des Végétaux, Unité de Mycologie, Domaine de Pixérécourt Bât. E, 54220 Malzéville, France; 2 Department of Agriculture, Food and Environment, University of Catania, Via Santa Sofia, 100, Catania, 95123 Italy; 3 Slovenian Forestry Institute, Department of Forest Protection, Večna pot 2, SI-1000 Ljubljana, Slovenia; 4 Forest Research, Alice Holt Lodge, Farnham, Surrey GU10 4LH United Kingdom; 5 Instituto Agroforestal Mediterráneo, Universitat Politècnica de València, Camino de Vera s/n, 46022 Valencia, Spain; 6 Instituto Nacional de Investigação Agrária e Veterinária I.P. (INIAV I.P.), Quinta do Marquês, 2780-159 Oeiras, Portugal; 7 Çankırı Karatekin University, Faculty of Forestry, 18200 Çankırı, Turkey; 8 Swiss Federal Institute for Forest, Snow and Landscape Research WSL, Zuercherstrasse 111, 8903 Birmensdorf, Switzerland; 9 Institute of Forestry and Rural Engineering, Estonian University of Life Sciences, 51006 Tartu, Estonia; 10 Julius Kühn-Institut, Institute for National and International Plant Health, Messeweg 11-12, 38104 Braunschweig, Germany; 11 Lithuanian Research Centre for Agriculture and Forestry, Vokė Branch, Zalioji Sq. 2, 02232 Vilnius, Lithuania; 12 Sustainable Forest Management Research Institute, University of Valladolid – INIA/Department of Vegetal Production and Forest Resources, University of Valladolid, 47011 Palencia, Spain; 13 Cardinal Stefan Wyszynski University in Warsaw, Faculty of Biology and Environmental Sciences, Wóycickiego 1/3 Street, 01-938 Warsaw, Poland; 14 Institute for Sustainable Plant Protection - National Research Council (IPSP-CNR), Via Madonna del Piano 10, I-50019 Sesto Fiorentino, Florence Italy; 15 Department for Innovation in Biological, Agro-food and Forest Systems (DIBAF), University of Tuscia, via S. Camillo de Lellis, snc, 01100 Viterbo, Italy; 16 Bioforest S.A. Camino a Coronel km 15S/N, 4030000 Concepción, Chile; 17 Department of Food, Environmental and Nutritional Sciences, University of Milan, via Celoria 2, I-20133 Milano, Italy; 18 Department of Biochemistry, Genetics and Microbiology, Forestry and Agricultural Biotechnology Institute (FABI), University of Pretoria, 0028 Hatfield, Pretoria, South Africa; 19 Department of Agricultural Sciences, Biotechnology, and Food Science, Cyprus University of Technology, 3036 Limassol, Cyprus; 20 Departamento de Biologia, CESAM, Universidade de Aveiro, 3810-193 Aveiro, Portugal; 21 Dipartimento di Scienze delle Produzioni Agroalimentari e dell’Ambiente (DISPAA), University of Florence, 50144 Florence, Italy; 22 Phytophthora Research Center, Department of Forest Protection and Wildlife Management, Faculty of Forestry and Wood Technology, Mendel University in Brno, Zemědělská 3, 613 00 Brno, Czech Republic; 23 Forest Health Center of Calabazanos, Regional Government of Castilla y León, JCyL, Poligono Industrial de Villamuriel, S/N, 30190, Villamuriel de Cerrato, Palencia, Spain; 24 Dipartimento di Scienze Agrarie, Alimentari e Forestali, Università degli Studi di Palermo, Viale delle Scienze, 90128 Palermo, Italy; 25 Plant Health & Environment Laboratory, Diagnostic and Surveillance Services, Biosecurity New Zealand, Ministry for Primary Industries, PO Box 2095, Auckland, 1140 New Zealand

**Keywords:** PCR-based techniques, Invasive species

## Abstract

*Fusarium circinatum* is a harmful pathogenic fungus mostly attacking *Pinus* species and also *Pseudotsuga menziesii*, causing cankers in trees of all ages, damping-off in seedlings, and mortality in cuttings and mother plants for clonal production. This fungus is listed as a quarantine pest in several parts of the world and the trade of potentially contaminated pine material such as cuttings, seedlings or seeds is restricted in order to prevent its spread to disease-free areas. Inspection of plant material often relies on DNA testing and several conventional or real-time PCR based tests targeting *F*. *circinatum* are available in the literature. In this work, an international collaborative study joined 23 partners to assess the transferability and the performance of nine molecular protocols, using a wide panel of DNA from 71 representative strains of *F*. *circinatum* and related *Fusarium* species. Diagnostic sensitivity, specificity and accuracy of the nine protocols all reached values >80%, and the diagnostic specificity was the only parameter differing significantly between protocols. The rates of false positives and of false negatives were computed and only the false positive rates differed significantly, ranging from 3.0% to 17.3%. The difference between protocols for some of the performance values were mainly due to cross-reactions with DNA from non-target species, which were either not tested or documented in the original articles. Considering that participating laboratories were free to use their own reagents and equipment, this study demonstrated that the diagnostic protocols for *F*. *circinatum* were not easily transferable to end-users. More generally, our results suggest that the use of protocols using conventional or real-time PCR outside their initial development and validation conditions should require careful characterization of the performance data prior to use under modified conditions (i.e. reagents and equipment). Suggestions to improve the transfer are proposed.

## Introduction

*Fusarium circinatum* Nelson Nirenberg & O’Donnell, formerly also known as *Gibberella circinata* Nirenberg & O’Donnell, is a harmful fungus causing pitch canker, a serious disease on pine trees. This pathogenic ascomycete attacks all *Pinus* species and *Pseudotsuga menziesii* (Mirb.) Franco, with varying levels of virulence^1–3^. All stages of pine are susceptible to the pathogen: seedling blight and damping-off, dieback of branches and stems on young and mature trees, where the most conspicuous symptoms are cankers accompanied by sometimes copious resin exudate (≪pitch canker≫)^4^. Dieback symptoms are also commonly observed in the crown due to the obstruction of water flow caused by the cankers and saturation of xylem by the excess resin produced by the tree. The multiplication of severe cankers on young or mature trees may lead to tree death^5^.

As for most tree pathogens, no economically and environmentally viable treatment is available to control or eradicate the fungus on a large scale. Management strategies are therefore focused on preventing the introduction of the pathogen, early detection and eradication of outbreaks in previously disease-free areas. *Fusarium circinatum* has been reported in different parts of the world, where it causes severe losses to the pine production industry (USA, South Africa, Korea, Japan, Spain) as well as in nurseries (Mexico, Haiti, Chile, Uruguay, Brazil, Colombia, South Africa) (EPPO Global database, https://gd.eppo.int/). In Europe, *F*. *circinatum* is officially present in Spain and Portugal in pine forests^6,7^, while it is also occasionally found in pine nurseries. The pathogen has also been found in French nurseries^8^, and in a public garden in Italy^9^, but it is currently considered as officially eradicated in both countries (EPP0 Global database, https://gd.eppo.int/). Since 2007, *F*. *circinatum* has been listed as a quarantine fungus for the EU, in order to prevent new introductions of infected material and further spread of the disease^10^. As a consequence of its quarantine status, a zero-tolerance policy is in force.

Management efforts should therefore focus on early detection of the pathogen in the different pathways of movement and introduction. Based on the pest risk assessment issued by the European Food and Environment Safety Agency (EFSA), the main pathways for the potential introduction of this fungus to disease-free areas are through contaminated pine seeds and seedlings^8^. To minimize the probability of an introduction and to reduce the cost associated with the eradication and control of this invasive pathogen, efficient management measures are needed^11^. For these reasons, the development of reliable diagnostic protocols is fundamental for the early and accurate detection of *F*. *circinatum* in pine-related commodities which can harbor and consequently spread the pathogen (i.e. substrates) such as seeds, seedlings, plants, young and mature trees. The diagnostic protocols should therefore be as specific and sensitive as possible^10,12^. False-negative detection results may lead to introduction of the pathogen in disease-free areas, while false-positive results may be responsible for unfair and inappropriate destruction of plant material, or a ban on trade with severe economic consequences.

Numerous diagnostic protocols targeting *F*. *circinatum* are currently available in the scientific literature and their use is suggested in international standard protocols by international bodies such as EPPO, ISTA or FAO-IPPC^13–15^. Despite most of them providing validation data supporting their accuracy, performance values are inconsistently reported or assessed in the original articles describing their development. Validation actions should be carried out to provide objective evidence that the test is suitable for the circumstances of use and can be considered for screening purposes^16^.

For instance, identification of *F*. *circinatum* may be achieved by isolation and morphological characterization of the pathogen, a common technique used in mycology. *Fusarium circinatum* displays several typical features, such as the presence of mono- and polyphialides as well as coiled sterile hyphae that aid diagnosis^17,18^. However, a recent study based on the phenotypical characteristics of isolates from a wide geographical range (Europe, America, Africa, and Asia) found that coiled sterile hyphae may not be a reliable morphological trait of *F*. *circinatum*, as previously reported^19^. Recent description of new *Fusarium* species in Colombia, which are also pine pathogens and can produce similar coiled hyphae in culture, further challenges the specificity of this morphology-based technique^20^. Additionally, morphological characterization is lengthy and requires considerable mycological expertise, whilst also not being efficient for the detection of quiescent forms of the pathogen that can be encountered in seedlings^21^ or in seeds.

A number of conventional and real-time polymerase chain reaction (PCR) assays targeting *F*. *circinatum* have been developed^13,22–28^, and another one is presented in the Supplementary Information section (Supplementary Information 1, hereafter referred to as Baskarathevan *et al*., unpublished). In the original articles, variable levels of validation are presented. For instance, the assessment of specificity and inclusivity used a more or less exhaustive range of *Fusarium* strains, depending on the availability of testing material, which included numbers of *F*. *circinatum* strains from different continents and newly described species, genetically related to *F*. *circinatum*. Considering the paramount importance of the reliability of a test when dealing with a quarantine pathogen, efforts should be focused on the continuous verification of the specificity of the protocols. This is particularly relevant when a *Fusarium* species is targeted, since this genus includes a steadily increasing number of newly described and cryptic species^20,29,30^. In addition, there is typically no data available to support the reliability of a given protocol when carried out in different laboratories, with different equipment and reagents than those described in the original papers. For instance, one may imagine that the specificity and the sensitivity of a test using conventional and real-time PCR may be altered by changing the brand of DNA polymerase enzyme, commercial ready-to-use master mix, or thermal-cycler/software. This in turn may affect the reliability of a diagnostic protocol targeting economically important pathogens such as *F*. *circinatum*^31,32^. Validation of diagnostic protocols is therefore a key element in establishing reference methods and to assess a laboratory’s competence and ability to produce reliable analytical data^12^.

The currently ongoing European COST action FP1406 “Pine Pitch Canker Strategies for Management of *Gibberella circinata* in Green Houses and Forests” (PINESTRENGTH) brings together 34 countries to establish a European-focused network dedicated to the Pine Pitch Canker pathogen. The main objectives are to increase knowledge on the biology, ecology and spread pathways of *F*. *circinatum*, to evaluate the potential for the development of effective and environmentally friendly prevention and mitigation strategies and to deliver these outcomes to stakeholders and policy makers. Early and accurate detection of *F*. *circinatum* is essential to achieve these goals. In this study, we compared for the first time performance of existing molecular tools targeting *F*. *circinatum*, with a wide range of DNA from target and non-target *Fusarium* species, in a large panel of 23 laboratories from European countries, South Africa and Chile. This collaborative study enabled us to provide useful data for the transferability of the different molecular-based diagnostic protocols. We propose recommendations for the preparation and use of future standard diagnostic protocols using conventional or real-time PCR assays.

## Results

### Indeterminate results

All 23 participants carried out the tests as requested. However, a few samples could not be tested by some of the participants due to loss or shortage of DNA template after multiple PCR run failures and were thus not considered in the analyses.

**Figure 1:**
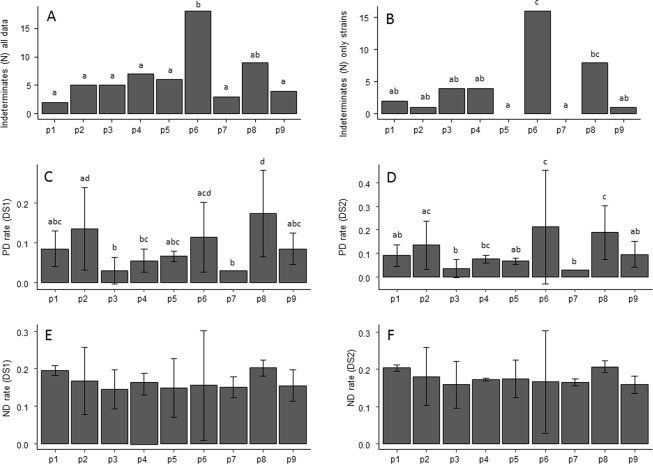
(**A**) Total number of indeterminate results by protocol considering all data (strains and inoculated seeds); (**B**) Total number of indeterminate results by protocol considering only strains (i.e. no inoculated seed data); (**C**) PD rate mean values and standard deviation by protocol for DS1 dataset; (**D**) PD rate mean values and standard deviation by protocol for DS2 dataset. (**E**) ND rate mean values and standard deviation by protocol for DS1 dataset; (**F**) ND rate mean values and standard deviation by protocol for DS2 dataset. The x – axis in all graphs represents the nine protocols tested in this study, from p1 to p9. Please refer to Table 4 for details of each protocol. Different letters indicate values are significantly different, according to Fisher’s Exact Test, for count data with simulated P-values based on 1e + 05 replicates.

All nine protocols generated indeterminate results (Table 1, Fig. 1A). The total number of indeterminate results was 58 (1.5% of the total analysis) and ranged from 0.42% for protocol p1 to 4.58% for protocol p6 (Table 1). The participants reported several reasons for rating results as indeterminate. These included late mean cycle threshold (Ct) values or inconsistent Ct values from the replicates for hydrolysis probe-based protocols, melting temperatures (Tm) and melting peaks slightly different from the positive control, or late mean Ct values for SYBR Green based protocols. Generally, laboratories performing conventional PCR protocols did not provide an explanation for indeterminate results. However, one participant sequenced each amplicon and rated the results as indeterminate when the sequence was not readable. Comparison of indeterminate results by Fisher’s exact tests revealed significant differences (P < 0.001) between protocols (Table 1, Fig. 1A). In particular, protocol p6 based on SYBR Green real-time PCR, yielded significantly more indeterminate results than most of the other protocols. In this case, the melting peak analysis required by protocol p6 often revealed the presence of melting peaks slightly different from the positive control, the presence of double melting peaks, or only one out of two replicates were positive for some of the DNA extracts.

**Table 1:** Performance values obtained during the collaborative study.

Protocol	End point PCR		SYBR Green real-time PCR			Hydrolysis probe real-time PCR				Significance of the difference between protocols
	p1	p9	p4	p5	p6	p2	p3	p7	p8	
Number of laboratories involved	6	6	6	5	4	6	6	4	6	
Number of samples analyzed and retained	474	472	473	393	393	474	474	316	473	
Number of indeterminate results^a,b^	2 A (2 AB)	4 A (2 AB)	7 A (4 AB)	6 A (0 A)	18 B (16 C)	5 A (1 AB)	5 A (4 AB)	3 A (0 AB)	9 AB (8 BC)	P < 0.001 (P < 0.001)
Negative Accord (NA)^b,c^	181 (180)	183 (179)	187 (183)	154 (154)	146 (130)	171 (171)	192 (191)	128 (128)	163 (161)	—
Positive Accord (PA)^b,c^	223 (221)	229 (231)	230 (227)	194 (189)	192 (191)	230 (226)	236 (232)	156 (153)	221 (220)	—
Negative Deviation (ND)^b,c^	53 (55)	43 (44)	45 (48)	34 (40)	35 (37)	46 (50)	40 (44)	28 (31)	55 (56)	—
Positive Deviation (PD)^b,c^	17 (18)	17 (19)	11 (15)	11 (11)	19 (35)	27 (27)	6 (7)	4 (4)	34 (37)	—
Diagnostic Sensitivity % (SE)^b,c^	80.8 (80.1)	84.3 (83.6)	83.6 (82.5)	85.0 (82.8)	84.0 (83.6)	83.3 (81.9)	85.5 (84.1)	84.8 (83.2)	80.1 (79.7)	P = 0.71 (P = 0.88)
Diagnostic Specificity % (SP)^b,c^	91.4 ABCD (90.9 AB)	92.4 ABCD (90.4 AB)	94.4 AC (92.4 AB)	93.3 ABC (93.3 AB)	88.5 ABD (78.8 C)	86.4 BD (86.4 AC)	97.0 C (96.6 B)	97.0 C (97.0 B)	82.6 D (81.6 C)	P < 0.001 (P < 0.001)
Diagnostic accuracy % (AC)^b,c^	85.2 AB (84.6 ABC)	87.7 AB (86.4 AB)	88.2 A (86.7 AB)	88.5 A (87.3 ACD)	85.9 AB (81.6 ABC)	84.6 AB (83.8 ABC)	90.3 A (89.2 B)	89.9 A (88.9 D)	81.2 B (80.5 CD)	P = 0.02 (P < 0.001)
Concordance level (%)^b^	96.8 A	93 C	96.8 A	93.7 C	74.6 D	88.5 B	93.2 C	97.7 A	93.5 C	P < 0.001
Positive deviation (PD)rate (%)^b,c^	8.6 ABC (9.1 AB)	8.5 ABC (9.6 AB)	5.6 BC (7.6 AB)	6.7 ABC (6.7 AB)	11.5 ACD (21.2 C)	13.6 AD (13.6 AC)	3.0 B (3.5 B)	3.0 B (3.0 B)	17.3 D (18.7 C)	P < 0.001 (P < 0.001)
Negative deviation (ND) rate %^b,c^	19.2 (19.9)	15.7 (16.1)	16.4 (17.5)	14.9 (17.5)	15.4 (16.2)	16.7 (18.1)	14.5 (15.9)	15.2 (16.8)	19.9 (20.3)	P = 0.716 (P = 0.89)

^a^Numbers outside brackets are the total number of indeterminate results, while those between brackets indicate the number of indeterminate results considering only pure *Fusarium* strain DNA (i.e. no DNA extracts from inoculated seeds).

^b^Values followed by the same letter in a line are not significantly different (P > 0.05).

^c^Numbers outside brackets are estimations for the DS1 dataset and those between brackets are estimations for the DS2 dataset.

When indeterminate results were analyzed with only pure *Fusarium* strain DNA (no DNA extracts from inoculated seeds), 37 indeterminate results were reported (63.8% of the total number of indeterminate results). Fisher’s exact tests revealed significant differences between protocols for these indeterminate results (P < 0.001, Table 1, Fig. 1B), and the rate of indeterminate ranged from 0.0% (protocol p5 and protocol p7) to 4.6% (protocol p6). In general, these indeterminate results mostly concerned non-target species (88.9% of the *Fusarium* strain DNA material indeterminate results).

Comparisons of indeterminate results between laboratories by protocol revealed significant differences for p2 (indeterminate data by laboratory ranging from 0% to 5.1%, P = 0.02), p5 (indeterminate data by laboratory ranging from 0% to 7.6%, P = 0.002), p6 (indeterminate data by laboratory ranging from 0% to 16.5%, P < 0.001), p8 (indeterminate data by laboratory ranging from 0% to 7.6%, P < 0.001) and p9 (indeterminate data by laboratory ranging from 0% to 5.1%, P < 0.001).

### Positive and negative deviation rates

All nine protocols exhibited positive deviations (or false positives) and negative deviations (or false negatives) regardless of the data set used (Table 1, Fig. 1C–F). Analysis of DS1 (the dataset for which an indeterminate result is ultimately determined to be the expected result, see methods section) revealed positive deviation rates (PD) ranging from 3.0% (protocol p7) to 17.3% (protocol p8) (Table 1, Fig. 1C). Comparison of PD by Fisher’s exact tests revealed significant differences (P < 0.001) between protocols (Table 1, Fig. 1C). Negative deviation rates (ND) in DS1 ranged from 14.5% (protocol p3) to 19.9% (protocol p8), but according to Fisher’s exact tests, the ND rates between protocols were not different (P = 0.71) (Table 1, Fig. 1E). Concerning DS2 (the dataset for which an indeterminate result is ultimately determined to be the contrary of the expected result, see methods section), PD ranged from 3.0% (protocol p7) to 21.2% (protocol p6) (Table 1, Fig. 1D). Significant differences between protocols for PD rates were observed according to Fisher’s exact tests (P < 0.001). ND in DS2 ranged from 15.9% (protocol p3) to 20.3% (protocol p8) (Table 1, Fig. 1F). ND comparisons between protocols using Fisher’s exact tests did not reveal any significant differences (P = 0.88).

Comparisons between laboratories using DS1 revealed significant PD rate differences only for p2 (PD rate by laboratory ranging from 3.0% to 27.3%, P = 0.007) and p8 (PD rate by laboratory ranging from 9.1% to 37.5%, P = 0.04). Concerning the ND rate in DS1, significant differences were observed in p2 (ND rate by laboratory ranging from 6.5% to 32.6%, P = 0.031) and p6 (ND rate by laboratory ranging from 4.3% to 40.9%, P < 0.001). When comparisons were performed using DS2, significant differences between laboratories were revealed for p2 (PD rate by laboratory ranging from 3.0% to 27.3%, P = 0.007) and p6 (PD rate by laboratory ranging from 6.1% to 63.6%, P < 0.001), for the PD rate, and for p6 (ND rate by laboratory ranging from 6.5% to 40.9%, P < 0.001) for the ND rate.

### Pattern of cross-reactions with non-target species

As shown previously, all protocols exhibited different levels of positive deviations (Table 1, Fig. 1C,D). Cross-reactions with DNA from strains of non-target *Fusarium* species were encountered for all nine protocols, but inconsistently between participating labs (Table 2). For many strains, a cros-s reaction with the DNA extract was reported for only a single participant among the four, five, or six laboratories involved, which corresponds to a unique reagent/equipment/operator combination.

**Table 2:** Detailed list of the false positive and false negative results obtained with the panel DNA from target and non-target species in this study.

Protocol number	Cross-reactions*	Indeterminate or false negative result
		(*F*. *circinatum* strain, originating country)**
**p1**	***F***. ***marasasianum*** **(5/6)**^**a**^	**NRRL26431, Japan (5/6)**
	***F***. ***pinninemorale*** **(5/6)**^**a**^	310/061, Spain (1/6)
	***F***. ***sororula*** **(5/6)**^**a**^	
	*F*. *temperatum* (2/6)^a^	
**p2**	***F***. ***begoniae*** **(6/6)**^**b**^	LSVM216, France (1/6)
	*F*. *concentricum* (1/6)^b^	LSVM1221, Spain (1/6)
	*F*. *culmorum* (1/6)^b^	FcCa01, Spain (1/6)
	***F***. ***fracticaudum*** **(3/6)**^**a**^	FcCa05, Spain (1/6)
	***F***. ***parvisorum*** **(3/6)**^**a**^	FcCa06, Spain (1/6)
	*F*. *pininemorale* (2/6)^a^	CSF-13, Spain (1/6)
	*F*. *sororula* (2/6)^a^	2306 BASA, Chile (1/6)
	***F***. ***subglutinans*** **(4/6)**^**b**^	
**p3**	*F*. *culmorum* (1/6)^b^	NRRL25708, USA (1/6)
	***F***. ***subglutinans*** **(3/6)**^**b**^	NRRL25333, S. Africa (1/6)
		FcCa06, Spain (1/6)
		CSF-18, Spain (1/6)
**p4**	*F*. *marasasianum* (1/6)^a^	
	*F*. *proliferatum* (1/6)^a^	
	***F***. ***subglutinans*** **(6/6)**^**b**^	
**p5**	*F*. *proliferatum* (1/5)^b^	CSF8, Spain (1/5)
	***F***. ***subglutinans*** **(6/5)**^**b**^	CSF10, Spain (1/5)
		CSF11, Spain (1/5)
**p6**	*F*. *acuminatum* (1/5)^a^	NRRL25708, USA (1/5)
	*F*. *fracticaudum* (1/5)^a^	NRRL25331, USA (1/5)
	*F*. *graminearum* (1/5)^b^	NRRL25333, S. Africa (1/5)
	*F*. *incarnatum* (1/5)^b^	FcCa02, Spain (1/5)
	*F*. *mangiferae* (1/5)^b^	FcCa05, Spain (1/5)
	***F***. ***marasasianum*** **(3/5)**^**a**^	FC042V, Spain (1/5)
	*F*. *parviporum* (1/5)^a^	FC035, Spain (1/5)
	*F*. *pininemorale* (1/5)^a^	CSF1, Spain (1/5)
	*F*. *reticulatum* (1/5)^a^	CSF2, Spain (1/5)
	*F*. *sacchari* (1/5)^b^	CSF3, Spain (1/5)
	*F*. *sororula* (1/5)^a^	CSF4, Spain (1/5)
	*F*. *sporotrichioides* (1/5)^b^	CSF7, Spain (1/5)
	***F***. ***subglutinans*** **(4/5)**^**b**^	CSF8, Spain (1/5)
	*F*. *torulosum* (1/5)^a^	CSF11, Spain (1/5)
	*F*. *thapsinum* (1/5)^b^	CSF12, Spain (1/5)
	*F*. *tricinctum* (2/5)^b^	CMW1219, S. Africa (1/5)
	*F*. *verticillioides* (1/5)^b^	
**p7**	***F***. ***temperatum*** **(4/4)**^**c**^	
**p8**	***F***. ***fracticaudum*** **(4/6)**^**a**^	**NRRL26431, Japan (6/6)**
	*F*. *incarnatum* (1/6)^a^	LSVM211, France (1/6)
	*F*. *mangiferae* (1/6)^a^	LSVM216, France (1/6)
	***F***. ***parvisorum*** **(4/6)**^**a**^	
	*F*. *proliferatum* (1/6)^a^	
	*F*. *sacchari* (1/6)^a^	
	***F***. ***sororula*** **(4/6)**^**a**^	
	***F***. ***subglutinans*** **(6/6)**^**a**^	
	***F***. ***temperatum*** **(5/6)**^**a**^	
	*F*. *verticillioides* (1/6)^a^	
**p9**	*F*. *begoniae* (1/6)^b^	
	*F*. *concentricum* (2/6)^b^	
	*F*. *culmorum* (1/6)^b^	
	***F***. ***fracticaudum*** **(5/6)**^**a**^	
	*F*. *fractiflexum* (1/6)^b^	
	*F*. *marasasianum* (1/6)^a^	
	*F*. *proliferatum* (1/6)^b^	
	*F*. *sororula* (1/6)^a^	
	***F***. ***subglutinans*** **(5/6)**^**b**^	
	*F*. *torulosum* (1/6)^a^	

*Number of participants for which a cross reaction was observed/number of participants involved. Species names in bold indicate a frequent cross-reaction was observed for the protocol with the 0.5 ng µL^−1^ DNA extract used.

**Number of participants for which the 0.5 ng µL^−1^ DNA extract of the *F*. *circinatum* strain was not picked up by the protocol or yielded an indeterminate result/number of participants involved.

^a^No reference sequence is available for this marker on GenBank for comparison with amplicon sequence, at the time of this study.

^b^Comparison of the amplicon sequence with IGS *F*. *circinatum* reference sequences available on GenBank shows several polymorphisms in the region between primers.

^c^Comparison of the amplicon sequence with TEF 1alpha *F*. *circinatum* reference sequences available on GenBank shows no polymorphism in the region between primers.

*Fusarium subglutinans* was the species that accounted for the most frequent cross-reactions, and its DNA yielded false-positive results for seven out of the nine protocols. Depending on the protocol, from three, up to six out of six laboratories observed cross-reaction with DNA of this species. *Fusarium temperatum* was the only, albeit consistent, cross-reaction observed with protocol p7. DNA from the newly-described species on pine from Colombia, i.e. *F*. *marasasianum*, *F*. *pinninemorale*, *F*. *sororula*, *F*. *fracticaudum*, and *F*. *parvisorum* also yielded frequent cross-reactions with four protocols (p1, p2, p8 and p9), and less frequently with protocol p6.

Reference sequences of target genes from species whose DNA cross-reacted with some of the PCR or real-time PCR tests were retrieved from GenBank and aligned with orthologous reference sequences of *F*. *circinatum*. The regions upstream and downstream of the forward and reverse PCR primers were removed. The alignments were manually scrutinized to check for the presence of interspecific polymorphism between the regions targeted by the primers. For some of the PCR or real-time PCR tests, it was shown that the presence of polymorphisms after sequencing the amplicon would be helpful to confirm the occurrence of cross-reactions, and possible false-positive results (Table 2).

### Inclusivity of the different protocols

In this work, inclusivity is defined as the ability of each protocol to detect DNA of the target species, regardless of the host plant, mating type, geographical origin and year of collection. Different patterns of inclusivity were observed between protocols. DNA from some of the *F*. *circinatum* strains yielded inconsistent negative results. Protocols p4, p7 and p9 successfully picked up all the 38 *F*. *circinatum* strains of both mating types included in the panel, regardless of the equipment, reagents and operator, thus supporting their excellent level of inclusivity. By contrast, protocols p1 and p8 almost systematically failed to yield positive results with DNA from the Japanese strain of *F*. *circinatum* NRRL2643. However, the rest of the false negative results were not reproducible between laboratories, and were only reported for one participant out of the four, five, or six involved, meaning that they were only observed for some operator/reagent/equipment combinations. Except for the Japanese strain of *F*. *circinatum* NRRL2643, these false negative results were observed with DNA from different *F*. *circinatum* strains originating from Spain, France, Chile, USA, and South Africa without any obvious pattern.

### Performance criteria and reproducibility

**Figure 2:**
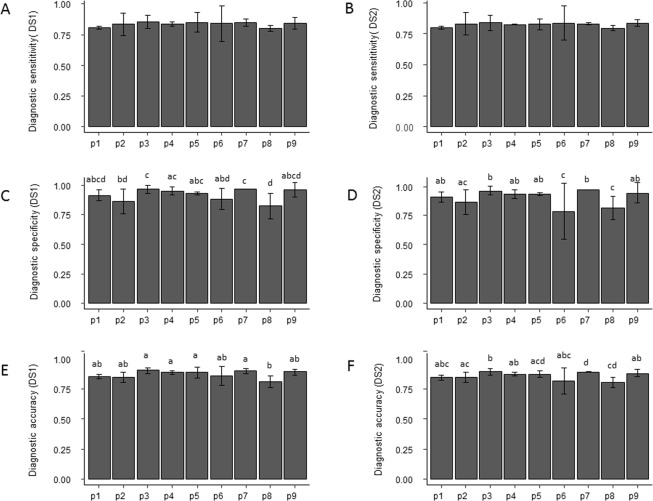
(**A**) Diagnostic sensitivity and standard deviation by protocol for the DS1 dataset. (**B**) Diagnostic sensitivity and standard deviation by protocol for the DS2 dataset. (**C**) Diagnostic specificity and standard deviation by protocol for the DS1 dataset. (**D**) Diagnostic specificity and standard deviation by protocol for the DS2 dataset. (**E**) Diagnostic accuracy and standard deviation by protocol for the DS1 dataset. (**F**) Diagnostic accuracy and standard deviation by protocol for the DS2 dataset. The x – axis in all graphs represents the 9 protocols tested in this study, from p1 to p9. Please refer to Table 4 for details of each protocol. Different letters indicate values are significantly different, according to Fisher’s Exact Test, for count data with simulated P-values based on 1e + 05 replicates.

Diagnostic sensitivity (SE) ranged from 80.1% (protocol p8) to 85.5% (protocol p3) and from 79.7% (protocol p8) to 84.1% (protocol p3) using the DS1 and DS2 datasets, respectively. Fisher’s exact tests did not reveal significant differences for SE between the nine protocols either for DS1 (P = 0.72) or DS2 (P = 0.88) (Table 1, Fig. 2A,B). By contrast, diagnostic specificity (SP) differed significantly, using both datasets (both P-values < 0.001). SP ranged from 82.6% (protocol p8) to 97% (for both protocols p3 and p7) in DS1 (Table 1, Fig. 2C). When SP was assessed for DS2, it ranged from 78.8% (protocol p6) to 97% (protocol p7) (Table 1, Fig. 2D). Significant differences in Diagnostic accuracy (AC) were observed between protocols for both datasets (P = 0.002 and P < 0.001 for DS1 and DS2, respectively). AC ranged from 81.2% (protocol p8) to 90.3% (protocol p3) in DS1 and from 80.5% (protocol p8) to 89.2% (protocol p3) in DS2 (Table 1, Fig. 2E,F). Concordance ranged from 74.6% to 97.7% for p6 and p7, respectively. Fisher’s exact tests revealed significant differences between methods (P < 0.001, Table 1).

### Analytical sensitivity

The analytical sensitivity was assessed for each protocol using serial dilutions of DNA from *P*. *pinaster* seeds spiked with *F*. *circinatum* conidia. However, inconsistent results were obtained with the serial dilutions. For example, when analyses were performed with the DS1 scenario, seven out of the nine evaluated protocols were able to detect at least one of the samples containing *F*. *circinatum* DNA in seeds at the highest concentration, i.e. 2 10^5^ conidia/mL of ground seed homogenate: p2, p3, p4, p5, p6, p7 and p9. Only p6 was able to give a positive result for all samples of this concentration. Similarly, these inconsistent results were also observed in data from DS2, and only p2, p3, p5 and p6 were able to detect at least one of the samples of the highest concentration of *F*. *circinatum* in seeds. This inconsistent behavior of inoculated material was observed for all of the serial dilution samples, in both datasets (Supplementary Dataset 1).

## Discussion

To establish an international surveillance network for the detection of outbreaks of pine pitch canker across Europe and other disease-free areas, harmonization of protocols for *F*. *circinatum* diagnosis is needed. To our knowledge, two international diagnostic protocols targeting this pathogen already exist. Nevertheless, one of them is a list of protocols that the international community has agreed are satisfactory, with minimal validation data available^13,15^ and the other relies solely on techniques such as mycological plating, that were selected because of their low cost and ease of implementation^14^. Most of the protocols targeting *F*. *circinatum* described in the literature lack a comprehensive evaluation of some basic performance criteria, such as specificity, inclusivity and sensitivity. Additionally, in most cases, specificity has not been re-evaluated in light of newly emerged or described *Fusarium* species occurring on *Pinus* spp^20^. In this study, we selected nine different detection protocols based on PCR and its variants. Their performance was assessed using a panel of 71 *Fusarium* strains and eight pine seed samples spiked with *F*. *circinatum* conidia. In order to achieve the best representation of *F*. *circinatum* strains (i.e. inclusivity), the panel included strains originating from six countries across four continents, and both mating types. Also included were recently-described *Fusarium* species isolated from pines, genetically related to *F*. *circinatum*, with overlapping morphological features, and some of them being pathogenic to *Pinus*^20^. This large panel of strains was used to assess the performance and transferability of the different protocols through an international collaboration involving a broad consortium of 23 partners. Each protocol was evaluated by a minimum of four, and up to six laboratories therefore ensuring a robust dataset. Practical and technical constraints led to an unbalanced number of laboratories involved per protocol, which means that some of the results should be read with care. In particular, although significant differences were observed for some criteria between participant laboratories and some of the protocols, caution is required before generalizing the results regarding non-significance of some of the statistical tests. Indeed, non-significant differences between protocols means non-identification of a difference rather than the absence of a difference altogether.

Our results showed that all protocols presented acceptable performance values in both datasets (>75%) for diagnostic accuracy, specificity and sensitivity, with some laboratories obtaining individual values close to 100% (Supplementary Dataset 1). Yet diagnostic specificity and accuracy differed considerably between protocols, irrespective of the technology involved (i.e. end-point PCR or real-time PCR using SYBR Green dye or specific hydrolysis probes). These differences were principally linked to cross-reactions with non-target species (positive deviations), and less commonly to consistent or erratic negative deviations with particular strains of *F*. *circinatum*.

The cross-reactions with DNA from non-target species observed in our study have not been reported in the original articles describing the protocols. We included a broader and more comprehensive panel of strains, revealing more information about the level of specificity of these protocols. However, our panel is not exhaustive and of course does not cover the entire biological range of genetically related *Fusarium* strains. Other unexpected cross-reactions may therefore occur, particularly with DNA from as yet undescribed *Fusarium* taxa. Cross-reactions were observed in all protocols, although at different levels, depending on the laboratory involved. Some of the erratic cross-reactions and false negative results may have occurred because of issues such as pipetting errors, DNA shearing, among others. However, some of the cross-reactions were more frequent and were due to lack of specificity/sensitivity of the molecular markers toward strains that had not been assessed during the original validation step of the original protocol by the authors, such as for genetically related *F*. *temperatum* and *F*. *subglutinans*. From a practical point of view, the presence of some of the *Fusarium* species whose DNA yielded false positive results are very unlikely on pine tissue, but the recent finding of Herron *et al*.^20^ showed that previously unknown species may be found on pine. In our experiment, a common DNA extraction procedure was followed for all the fungal strains, which sometimes differed from the original article describing each of the nine protocols. It cannot be ruled out that the DNA extraction procedure used in our study had an effect of PCR or real-time PCR specificity or inclusivity. Another aspect to consider is that for the sake of harmonization, a standard concentration of *Fusarium* DNA was used throughout the study (0.5 ng/µL). This may not always reflect the actual concentration that may occur when testing real pine samples contaminated with these *Fusarium* species, and the likelihood of cross-reactions with non-target DNA probably increases with higher concentrations. At the same time, certain strains of *F*. *circinatum* were ‘missed’ by some of the protocols (especially the *F*. *circinatum* strain from Japan), with false-negative results that were not reported in the original articles, except by Ramsfield *et al*. (2008) regarding protocol p1. This suggests that some of the *F*. *circinatum* strains travelling with plant material such as seeds might not be detected when using some of the protocols assessed here. Our data provide a first evaluation of the inclusivity of nine protocols, which can be useful for laboratories in charge of official analyses, by elucidating the level of uncertainty associated with some of the protocols used throughout the world. Although diagnostic specificity across all protocols was rather high (>75%), no protocol was 100% specific with the present panel of *F*. *circinatum* strains. These false-positives may not be acceptable when dealing with a pathogen subjected to strict phytosanitary regulations.

Concordance varied between protocols and ranged from 74.6 to 97.7%. Analysis of the differences between laboratories that tested the same protocol also showed that indeterminate results and negative and positive deviations differed significantly. These results suggest that molecular detection methods may not always be easily transferable. Basically, they clearly illustrate that deviations from the “original recipe”, i.e. the use of different equipment, consumables, but also operators, might compromise the stringency of the reactions, and therefore the specificity of the results. This is particularly important when dealing with a quarantine pathogen, with a zero-tolerance policy. Positive deviations may lead to the inappropriate destruction of goods, whereas negative deviations could fail to prevent introduction of the pathogen into disease-free areas.

We also showed that all the protocols exhibited some problems in result interpretation (indeterminate results), independently of the PCR technology used. However, end-point PCR generally yielded fewer indeterminate results, across all partners, probably linked to the simplicity of result interpretation, based on the observation of a band on an electrophoresis gel, with little room for doubt. Concerning the other PCR techniques, it can be suggested that interpretation of the melting curves was not straightforward when using the SYBR Green real-time PCR, and setting the fluorescence threshold for the calculation of the cycle threshold value when using a hydrolysis probe was sometimes done inconsistently between partners. In addition to this problem of result interpretation, some of the protocols required particular settings that may not have worked well under different conditions. For example, protocol p2^24^ requires an unusually high hybridization temperature of 70 °C to ensure specificity, which seemed to cause a sensitivity problem when used in certain laboratories or with different reagents/equipment than the ones originally described. In this work, the statistical analysis of indeterminate results was enabled by processing data under two scenarios (DS1 and DS2). In all cases results from both datasets were consistent, leading consequently to the same conclusions. This is an important point because it means that differences in performance criteria between protocols were not influenced by indeterminate results, which represented less than 2% of the total results.

We did not evaluate and compare the sensitivity of a protocol based on mean Ct values, but rather on its ability to yield positive results with lower target concentrations. This approach enabled comparison of conventional PCR, for which no quantitative results are generated with real-time PCR protocols. Additionally, we chose not to provide cutoff values because the sensitivity of a test should not be dependent on Ct values, but rather on its ability to reliably amplify and detect a low concentration of target DNA^33^. Late Ct values may still be valid and confidently used if the test specificity has been correctly designed and evaluated^34^. Despite protocols p2, p3, p5 and p6 consistently yielding positive results for the highest conidia spiking quantity, the data of our study showed that positive results for lower concentrations were rarely obtained. This made it very difficult to compare the protocols to each other regarding analytical sensitivity with seeds. However, using protocols p2 and p6, successful detection of *F*. *circinatum* in naturally infected pine seeds has been reported by Ioos *et al*.^24^ and Dreaden *et al*.^25^, and protocol p2 has been used for years in ANSES for the interception of naturally infected imported seed lots (Guinet, C., ANSES, pers. Comm.). This suggests that the modified method used in our study for the preparation of artificially infected seed DNA was not able to provide samples with a sufficient level of contamination, probably inferior to what is expected with real-world samples. In this respect, a preliminary biologic enrichment of the seed in a broth of culture medium seems a very efficient method to improve detection of *F*. *circinatum* in seeds at low levels^13^.

One of the main recommendations resulting from the present study is that the transferability of a PCR or real-time PCR protocol should be thoroughly and continuously assessed before becoming a standard. In other words, the ability to yield accurate results when used under slightly different conditions should be checked. Indeed, it is very unlikely that all the specific brands of reagents and equipment described in the original scientific papers are available to end-users. In this study, partner laboratories were free to use their own real-time equipment and brand of reagents, such as DNA polymerases, PCR or real-time PCR master mixes. Discrepancies of results between laboratories regarding false positive and false negative rates, as well as the different analytical sensitivities confirm that changing the brand and type of DNA polymerase^35^ and equipment^31,36^ may affect the reliability of the results. Changing the DNA polymerase may also generate the amplification of non-specific amplicons, especially when working with symptomatic pine DNA extracts (Piškur, B., pers. comm.). This observation is in line with Bustin & Huggett^33^ who showed that the performance of a real-time PCR assay varied with different master mixes, probably due to differences in Mg^2+^ concentrations and the addition of undisclosed stabilizers to the buffer affecting primer and probe annealing.

Another parameter to be considered is that interpretation of the fluorescence levels yielded in the real-time PCR reactions requires the enforcement of decision rules, either by the operator, or the analysis software. In turn, the decision to rate a Ct value as a positive or indeterminate result may be influenced by internal rules, which are not the same between laboratories. In addition, slight variations induced by the operator or the equipment, such as pipetting errors, temperature drift or thermic heterogeneity of the thermal cycler block may have an effect on the stringency of the PCR reaction and thus, in turn may affect the analytical specificity and sensitivity^33,37–39^.

In line with other guidelines proposed for testing genetically modified organisms^40^, we therefore recommend that a preliminary assessment of the robustness and transferability of a new protocol should be carried out to provide an indication of its performance under different conditions than the ones used during its development. This assessment should be carried out in addition to the classical performance criteria assessed during the initial validation process. This may be achieved by, for instance, the organization of a collaborative study, using a large and representative panel of target and non-target taxa, and involving as many different reagent brands and thermal cycler types as possible. Therefore, the end users should bear in mind that the performance data of a conventional or real-time PCR protocol described in the original articles are intimately linked to the reagents, equipment and decision rules used. It is strongly suggested that individual laboratories should carry out their own characterization if these parameters are modified, and even if no parameter is modified at all. To this aim, a series of “reference samples” should be maintained and provided by a “reference laboratory” to any laboratory intending to establish and maintain an accurate diagnostic test^41^. The organization of training sessions by these reference laboratories would also help to share experience and knowledge about the use of a given protocol, and would harmonize the practices and decision rules. This is of paramount importance when targeting a quarantine pathogen, for which very strict regulations are enforced.

We also advocate the continuous verification of the specificity of published protocols, in order to consider new taxa that are continuously described in the literature. This can be achieved by *in silico* evaluation, by blasting the primer and probe sequences on international DNA databases such as GenBank on a regular basis, and by wet lab testing of newly described strains. Another suggestion to ensure the accuracy of the positive results is to analyze the amplicon sequence and/or to use additional tests targeting other loci in the genome of the target organism. Currently, the two international protocols for the diagnosis of *F*. *circinatum*^13,15^ recommend sequencing of the amplicon after a positive result via conventional or SYBR Green real-time PCR using the CIRC1A-CIRC4A primers^22^. However, our study suggests that a similar procedure should also apply for the other available protocols targeting *F*. *circinatum*, even for those using hydrolysis probe-based methods. It is advisable that such a complementary approach should be followed to verify results of particular importance such as first reports in disease-free areas. For some of the protocols, analysis of the amplicon sequence, trimmed from the primers’ sequences may help confirm the accuracy of the result. However, sequencing will not always be sufficient. Firstly, this is dependent on reference sequences being available in databases. Secondly, this approach will not work if undescribed species share a 100% match to *F*. *circinatum* (see Table 2 footnotes). Lastly, confirmation by sequencing is not always possible when the conventional or real-time PCR test targets a region of unknown function such as a Sequence Characterized Amplified Region (SCAR). Hence, no orthologous sequences for other *Fusarium* species are available for the SCAR targeted by protocol p1 and p8.

In addition, positive samples could be further processed in order to isolate the pathogen in pure culture, allowing the identification of the pathogen by both morphological and molecular features^42^. Combining molecular and morphological data would of course secure identification of the pathogen, particularly important for first reports, and will help to increase knowledge of the morphologic and genetic diversity of the pathogen. In this respect, it is necessary to establish protocols providing a representative sampling strategy, starting from plant tissue. With the exception of seeds, for which a strategy has been proposed^43^, there is to our knowledge no standard for sampling plants or adult trees, tackling for instance the minimum number of samples that should be taken for assuring the absence of the pathogen, irrespective of the analysis technique chosen (molecular or isolation).

## Methods

### Participants and selection of protocols

An official call for participation was issued in 2016 in the framework of the COST action FP1406 PINESTRENGTH. In all, 23 laboratories representing 18 countries participated in the study (Table 3). Only laboratories with sufficient experience in molecular biology-based detection techniques and appropriate equipment were involved. Samples were sent to each participant on June 7^th^, 2017. The analyses were to be completed and results returned to the organizer by the end of August 2017.

**Table 3:** Partners involved in the collaborative project.

Partner Label	Institute/laboratory	Description	Country, city
1	Sustainable Forest Management Institute	University of Valladolid	Spain, Palencia
2	Institute of Forestry and Rural Engineering	Estonian University of Life Sciences	Estonia, Tartu
3	Institute for National and International Plant Health	Julius Kühn-Institute	Germany, Braunschweig
4	Vokė Branch, Lab of Biotechnology	Lithuanian Research Centre for Agriculture and Forestry	Lithuania, Vilnius
5	Department for Innovation in Biological, Agro-food and Forest Systems (DIBAF),	University of Tuscia	Italy, Viterbo
6	Instituto Agroforestal Mediterráneo	Universitat Politècnica de València	Spain, Valencia
7	Laboratoire de la santé des végétaux	French Agency for food, environmental and occupational health safety (ANSES)	France, Malzéville
8	Faculty of Biology and Environmental Sciences	Cardinal Stefan Wyszynski University in Warsaw	Poland, Warsaw
9	Forest Health and Biotic Interactions/Phytopathology	Swiss Federal Institute for Forest, Snow and Landscape Research WSL	Switzerland, Birmensdorf
10	Instituto Nacional de Investigação Agrária e Veterinária I.P.,	State Laboratory of the Ministry of Agriculture, Forests and Rural Development	Portugal, Oeiras
11	Institute for Sustainable Plant Protection	National Research Council	Italy, Florence
12	Forest Research	Forestry Commission	United Kingdom, Farnham
13	Centro de Biotecnología	Universidad de Concepción	Chile, Concepción
14	Department of Food, Environmental and Nutritional Sciences	University of Milan	Italy, Milan
15	Forestry and Agricultural Biotechnology Institute	University of Pretoria	South Africa, Pretoria
16	Department of Agricultural Sciences, Biotechnology and Food Science	Cyprus University of Technology	Cyprus, Limassol
17	Department of Biology	University of Aveiro	Portugal, Aveiro
18	Dipartimento di Scienze delle Produzioni Agroalimentari e dell’Ambiente (DISPAA)	University of Florence	Italy, Florence.
19	Phytophthora Research Centre	Mendel University in Brno	Czech Republic, Brno
20	Laboratory of Forest Protection	Slovenian Forestry Institute	Slovenia, Ljubljana
21	Forest Health Center of Calabazanos	Regional Government of Castilla y León (JCyL)	Spain, Palencia
22	Faculty of Forestry, Forest Pathology Laboratories	Applied Sciences University of Isparta	Turkey, Isparta
23	Department of Agriculture, Food and Environment	University of Catania	Italy, Catania

At the time the project was started, a total of nine conventional or real-time PCR protocols targeting *F*. *circinatum* (p1 to p9) were available in the literature or were brought to our knowledge (Table 4). Protocols included several formats of PCR amplification and labeling. Protocols p1 and p9 use conventional or end-point PCR^13,23^, p4, p5, and p6 use SYBR Green-based real-time PCR^22,25,26^, and p2, p3, p7, and p8 use real-time PCR hydrolysis probe-based tests^24,27,28^. In order to balance the comparison among the protocols and to provide a sufficient amount of data to compute the performance criteria, each protocol was assessed by at least four different partners.

**Table 4:** List of *F*. *circinatum* diagnostic protocols assessed during the collaborative study.

Protocol number	Reference	Target in the *F*. *circinatum* genome	Type of assay
p1	Ramsfield *et al*.^23^	Two independent sequence characterized amplified regions (SCAR)	End-point PCR
p2	Ioos *et al*.^24^	rDNA Intergenic spacer (IGS)	Real-time PCR with hydrolysis probe
p3	Lamarche *et al*.^27^	rDNA Intergenic spacer (IGS)	Real-time PCR with hydrolysis probe
p4	Schweigkofler *et al*.^22^	rDNA Intergenic spacer (IGS)	Real-time PCR with SYBR Green staining
p5	Fourie *et al*.^26^	rDNA Intergenic spacer (IGS)	Real-time PCR with SYBR Green staining
p6	Dreaden *et al*.^25^	rDNA Intergenic spacer (IGS)	Real-time PCR with SYBR Green staining
p7	Luchi *et al*.^28^	Translation elongation factor 1-alpha gene (TEF)	Real-time PCR with hydrolysis probe
p8	Baskarathevan *et al*. (Supplementary Information 1)	Sequence characterized amplified region (SCAR)	Real-time PCR with hydrolysis probe
p9	EPPO^13^, appendix 4	rDNA Intergenic spacer (IGS)	End-point PCR

Protocols were conducted following the description in the original article, observing the amplification parameters (cycling conditions, temperatures settings) and reaction mixtures (primers and probe concentrations, reaction and DNA template volumes) indicated by the authors and summarized in a reference document that was sent to each participant along with the DNA samples (Supplementary Information 2). However, if not available in the participant laboratory, the DNA polymerase or commercial real-time PCR master mix described in the original articles were replaced by the reagents typically used by the participant laboratory (for further information refer to Supplementary Information 3). Each participating laboratory was free to use its own PCR equipment.

### *F*. *circinatum*-specific primers and probes

Each partner provided the primers for the protocols using end-point PCR and SYBR Green real-time PCR, as described in the original articles. To cut down costs, the primers/probe combinations required for the different hydrolysis probe real-time PCR protocols were only purchased once by one of the partners and distributed to all the participants as ready-to-use aliquots of 30 µM (primers) or 10 µM (probe) solutions, in 1.5 mL amber microtubes. Primers/probe combinations for p2 and p7, p3, and p8 were custom made by Eurogentec (Seraing, Belgium), Integrated DNA technology (Skokie, Illinois), and Biosearch Technologies (Petaluma, California), respectively. Primers and probes were shipped at room temperature by a fast delivery service and kept in a freezer until used for testing.

### Fungal strains and preparation of panels of DNA samples

A panel of 71 monosporic *Fusarium* spp. strains representing 29 distinct species was used (Table 5). Species identity was confirmed by EF1 alpha gene sequencing^44^, if the strain was not obtained from an international fungal collection. It included 38 *F*. *circinatum* strains from different geographical origins, mating types, host tree species and environments, thus covering as much of the genetic diversity of the pathogen as possible. Thirty-three other *Fusarium* strains were also included. They represented species that are either genetically close to *F*. *circinatum*, or inhabit the same ecological niche, i.e. pine woody tissue, pine seeds, pine roots, or soil. Also included were recently described species of *Fusarium* associated with pine cankers in Colombia, i.e. *F*. *parvisorum*, *F*. *sororula*, *F*. *marasasianum*, *F*. *pininemorale*, and *F*. *fracticaudum*^20^. The strains were sent from different providers, and were kept on agar slants at 5 °C before handling. As *F*. *circinatum* is considered a quarantine organism for the European Union (EU), all strains from this species were maintained and manipulated in level 3 biohazard containment facilities in ANSES Plant Health Laboratory (here named as ANSES), in Malzéville, France, in compliance with EU Directive 2008/61/EC. Taking into consideration that the *Fusarium* strains from Colombia are recently described species^20^ not found in the EU, it was decided to manipulate them under the same conditions as *F*. *circinatum*.

**Table 5:** List of *Fusarium* spp. strains used in the collaborative study.

Code	Species	Strain	Mating type	Host	Origin	Environment	Provider
F1	*F*. *circinatum*	LSVM211	MAT-1	*P*. *menziesii*	France (Perpignan)	Private garden	R. Ioos
F2	*F*. *circinatum*	LSVM216	MAT-2	*P*. *radiata*	France (Vendée)	Nursery	R. Ioos
F3	*F*. *circinatum*	LSVM217	MAT-2	*P*. *radiata*	France (Côtes d’Armor)	Nursery	R. Ioos
F4	*F*. *circinatum*	LSVM1221	MAT-2	*P*. *radiata*	Espagne (Basque country)	Forest	J. Aguayo
F5	*F*. *circinatum*	NRRL26431	MAT-1	unkn.	Japan	unkn.	K. O’Donnell
F6	*F*. *circinatum*	NRRL25708	MAT-1	unkn.	USA	unkn.	K. O’Donnell
F7	*F*. *circinatum*	NRRL25331	MAT-1	unkn.	USA	unkn.	K. O’Donnell
F8	*F*. *circinatum*	NRRL25333	MAT-2	unkn.	S-Africa	unkn.	K. O’Donnell
F11	*F*. *circinatum*	FcCa01	MAT-2	*P*. *radiata*	Spain (Cantabria, Rionansa)	Forest	J. Diez
F12	*F*. *circinatum*	FcCa02	MAT-2	*P*. *radiata*	Spain (Cantabria, Castrourdiales)	Forest	J. Diez
F13	*F*. *circinatum*	FcCa05	MAT-2	*P*. *radiata*	Spain (Cantabria, Mazcuerras)	Forest	J. Diez
F14	*F*. *circinatum*	FcCa06	MAT-2	*P*. *radiata*	Spain (Cantabria, Comillas)	Forest	J. Diez
F15	*F*. *circinatum*	FC042v	MAT-2	*P*. *radiata*	Spain (Cantabria, Cabezón de la Sal)	Forest	J. Diez
F16	*F*. *circinatum*	FC035	MAT-2	*P*. *radiata*	Spain (Cantabria, Cabezón de la Sal)	Forest	J. Diez
F17	*F*. *circinatum*	CSF-1	MAT-1	*P*. *pinea*	Spain (Burgos)	Reforestation seedling	A. Sanz-Ros
F18	*F*. *circinatum*	CSF-2	MAT-1	*P*. *radiata*	Spain (León)	Insect (*Brachyderes* sp.)	A. Sanz-Ros
F19	*F*. *circinatum*	CSF-3	MAT-1	*P*. *radiata*	Spain (León)	Seed (cones)	A. Sanz-Ros
F20	*F*. *circinatum*	CSF-4	MAT-1	*P*. *radiata*	Spain (León)	Forest (twig)	A. Sanz-Ros
F22	*F*. *circinatum*	CSF-6	MAT-1	*P*. *radiata*	Spain (León)	Forest (stem)	A. Sanz-Ros
F23	*F*. *circinatum*	CSF-7	MAT-2	*P*. *radiata*	Spain (León)	Forest (stem)	A. Sanz-Ros
F24	*F*. *circinatum*	CSF-8	MAT-2	*P*. *nigra*	Spain (Palencia)	Reforestation seedling	A. Sanz-Ros
F26	*F*. *circinatum*	CSF-10	MAT-1	*P*. *nigra*	Spain (León)	Reforestation seedling	A. Sanz-Ros
F27	*F*. *circinatum*	CSF-11	MAT-1	*P*. *nigra*	Spain (Valladolid)	Nursery	A. Sanz-Ros
F28	*F*. *circinatum*	CSF-12	MAT-1	*P*. *sylvestris*	Spain (Valladolid)	Nursery	A. Sanz-Ros
F29	*F*. *circinatum*	CSF-13	MAT-2	*P*. *pinaster*	Spain (Valladolid)	Seeds	A. Sanz-Ros
F30	*F*. *circinatum*	116	MAT-2	*P*. *nigra*	Spain (Galicia)	Nursery	M. Berbegal
F31	*F*. *circinatum*	164	MAT-1	*P*. *sylvestris*	Spain (Asturias)	Nursery	M. Berbegal
F32	*F*. *circinatum*	221	MAT-2	*P*. *radiata*	Spain (Cantabria)	Nursery	M. Berbegal
F33	*F*. *circinatum*	253	MAT-1	*P*. *nigra*	Spain (Galicia)	Nursery	M. Berbegal
F34	*F*. *circinatum*	822	MAT-1	*P*. *pinaster*	Spain (Galicia)	Seeds	M. Berbegal
F35	*F*. *circinatum*	07/0649 1b	MAT-1	*P*. *pinaster*	Spain (Asturias)	Nursery	M. Berbegal
F36	*F*. *circinatum*	310/061	MAT-1	*P*. *palustris*	Spain (Asturias)	Nursery	M. Berbegal
F37	*F*. *circinatum*	2028	MAT-2	*P*. *radiata*	Chile	Nursery	R. Ahumada
F38	*F*. *circinatum*	2738	MAT-2	*P*. *radiata*	Chile	Nursery	R. Ahumada
F39	*F*. *circinatum*	INV19	MAT-2	*P*. *radiata*	Chile	Nursery	R. Ahumada
F40	*F*. *circinatum*	2306 BASA	MAT-2	*P*. *radiata*	Chile	Nursery	R. Ahumada
F41	*F*. *circinatum*	CMW 1219	MAT-2	*Pinus* sp.	South Africa	unkn.	MJ. Wingfield (FABI)
F42	*F*. *circinatum*	CMW 350	MAT-1	*Pinus* sp.	USA (California)	unkn.	MJ. Wingfield (FABI)
F51	*F*. *begoniae*	LSVM293	MAT-1	*Begonia elatior*	France		R. Ioos
F52	*F*. *concentricum*	NRRL 25181		unkn.	France		K. O’Donnell
F53	*F*. *fujikuroi*	LSV667	MAT-2	*Zea mays*	France		R. Ioos
F54	*F*. *mangiferae*	NRRL25226	MAT-2	unkn.	unkn.		K. O’Donnell
F55	*F*. *nygamai*	NRRL13448		unkn.	unkn.		K. O’Donnell
F56	*F*. *proliferatum*	LSVM673	MAT-2	*Populus* sp.	France		R. Ioos
F57	*F*. *sacchari*	NRRL13999		unkn.	unkn.		K. O’Donnell
F58	*F*. *subglutinans*	LSVM869	MAT-1	*Zea mays*	France		R. Ioos
F59	*F*. *subglutinans*	LSVM704	MAT-1	*Zea mays*	France		R. Ioos
F60	*F*. *temperatum*	LSVM870	MAT-2	*Zea mays*	France		R. Ioos
F61	*F*. *thapsinum*	NRRL22045		unkn.	unkn.		K. O’Donnell
F62	*F*. *verticillioides*	LSVM873		*Zea mays*	France		R. Ioos
F63	*F*. *verticillioides*	437-6		*Glycine max*	Italy		M Pasquali
F64	*F*. *fractiflexum*	NRRL28852	MAT-2	unkn.	unkn.		K. O’Donnell
F65	*F*. *proliferatum*	FGSC 7421	MAT-2	unkn.	unkn.		J.F. Leslie
F66	*F*. *parvisorum*	CMW 25267	MAT-2	*Pinus patula*	Columbia	Commerial nursery	MJ. Wingfield (FABI)
F67	*F*. *sororula*	CMW 25254	MAT-2	*Pinus* spp.	Columbia	Commerial nursery	MJ. Wingfield (FABI)
F68	*F*. *marasasianum*	CMW 25261	MAT-2	*Pinus patula*	Columbia	Commerial nursery	MJ. Wingfield (FABI)
F69	*F*. *pininemorale*	CMW 25243	MAT-1	*Pinus tecunumanii*	Columbia	Plantation	MJ. Wingfield (FABI)
F70	*F*. *fracticaudum*	CMW 25245	MAT-2	*Pinus maximinoi*	Columbia	Plantation	MJ. Wingfield (FABI)
F72	*F*. *avenaceum*	Do_US_Nat_2_1		seed of *Douglasia* sp.	USA		WSL - Phytopathology
F73	*F*. *incarnatum-equiseti species complex*	Do_US_Nat_3_1		seed of *Douglasia* sp.	USA		WSL - Phytopathology
F74	*F*. *sporotrichioides*	Do_US_Nat_32_1		seed of *Douglasia* sp.	USA		WSL - Phytopathology
F75	*F*. *tricinctum species complex*	Do_US_Sno_49_1		seed of *Douglasia* sp.	USA		WSL - Phytopathology
F76	*F*. *acuminatum*	Do_US_VC_49_1		seed of *Douglasia* sp.	USA		WSL - Phytopathology
F77	*F*. *torulosum*	Do_US_VC_5_1		seed of *Douglasia* sp.	USA		WSL - Phytopathology
F78	*F*. *graminearum*	Do-Mur/17-1		seed of *D*. *menziesii*	USA		WSL - Phytopathology
F79	*F*. *proliferatum*	FI-BOS/13-1		seed of *Picea* sp.	Switzerland		WSL - Phytopathology
F80	*F*. *reticulatum negundis*	FI-BOS/14-1		seed of *Picea* sp.	Switzerland		WSL - Phytopathology
F81	*F*. *redolens*	Do-D/11-1		seed of *Douglasia* sp.	Switzerland		WSL - Phytopathology
F82	*F*. *culmorum*	CSF-14		*Pinus pinea*	Spain (Palencia)	reafforestation seedling	A. Sanz-Ros
F83	*F*. *torulosum*	CSF-15		*Pinus nigra*	Spain (León)	reafforestation seedling	A. Sanz-Ros
F84	*F*. *oxysporum*	CSF-16	MAT-2	*Pinus pinea*	Spain (Palencia)	reafforestation seedling	A. Sanz-Ros
F85	*P*. *pinaster* seed spiked with 10^5^ conidia of strain F7					—	—
F86	*P*. *pinaster* seed spiked with 10^4^ conidia of strain F7					—	—
F87	*P*. *pinaster* seed spiked with 10^3^ conidia of strain F7					—	—
F88	*P*. *pinaster* seed spiked with 10^2^ conidia of strain F7					—	—
F89	*P*. *pinaster* seed spiked with 10^5^ conidia of strain F11					—	—
F90	*P*. *pinaster* seed spiked with 10^4^ conidia of strain F11					—	—
F91	*P*. *pinaster* seed spiked with 10^3^ conidia of strain F11					—	—
F92	*P*. *pinaster* seed spiked with 10^2^ conidia of strain F11					—	—

To avoid biases generated by the involvement of different operators and laboratories and to minimize the risk of moving around living *F*. *circinatum* strains, all participants worked with DNA extracts rather than with living cultures. All strains were first gathered in ANSES and kept on site. For DNA extraction, the strains were first cultured on potato dextrose liquid media (PD Broth, DIFCO™), for approximately 5 days, after which 100 to 200 mg of fresh mycelium was harvested. Genomic DNA (gDNA) was extracted using the DNeasy plant mini kit™ (Qiagen, Courtaboeuf, France) following the manufacturer’s guidelines, after grinding mycelium with a Lysis matrix A tube containing one 6-mm ceramic sphere and garnet matrix (MP Biomedicals, Santa Ana, CA, USA) and homogenized for 20 s at 6.5 U (m/sec) using a FASTprep 24 device (MP Biomedicals). DNA concentration was estimated using the Nanodrop 2000 Spectrophotometer™. For each strain, genomic DNA was produced from biological replicates and mixed/homogenized in order to obtain enough DNA to be tested in all of the different protocols and to supply to all of the partners. For each strain, the quality of the DNA extract was assessed by successful PCR amplification of the Internal Transcribed Spacer rDNA using the ITS1/ITS4 primer pair^45^, and the DNA concentration normalized to 0.5 ng µL^−1^ and distributed as 50-µL aliquots in individual 2-mL microtubes (F1 to F84, Table 5). In total, each laboratory received 38 *F*. *circinatum* DNA samples (target DNA) and 33 non-target DNA samples. All samples were anonymously labeled, shipped at room temperature by fast delivery service, and kept in a freezer until analysis.

A set of DNA from *F*. *circinatum*-artificially infected pine seeds was also prepared. Two strains of *F*. *circinatum* (F7 and F11) were cultured for 6 days at 22 °C under cool-white fluorescent lights with a 12-h light period on Spezieller Nährstoffarmer Agar (SNA) medium to allow macro- and microconidia production^46^. Microconidia were harvested by washing the surface of cultures with 10 mL of deionized sterile water with 0.01% Tween 20. The resulting suspension was diluted with sterile water to obtain a final concentration of 2.52 × 10^3^ and 1.90 × 10^3^ conidia µL^−1^ for F7 and F11, respectively, based on counts made using a hemocytometer. Healthy *Pinus pinaster* seeds were first incubated in liquid PD Broth media as described by Ioos *et al*.^24^, in order to simulate biological enrichment of natural samples, as is performed in routine detection analysis. Four replicates of one thousand healthy seeds each were incubated for 72 h at 22 °C in a sterile Easy flat flask containing 50 mL of PD Broth. After incubation, the contents of the Easy flask (healthy seeds + liquid medium) were aseptically transferred into a sterilized grinding bowl, and ground for 1 min using a Microtron MB 550 mixermill (Kinematica, Lucerne, Switzerland). Seventy subsamples of 500 µL of homogenate were collected using a micropipette and transferred into individual sterile 2-mL microtubes. For each of the two *F*. *circinatum* strains, 4 sets of 14 homogenized healthy seed subsamples were spiked with 1 × 10^5^, 1 × 10^4^, 1 × 10^3^, and 1 × 10^2^ conidia, respectively. One set was spiked with sterile water to be used as negative control for the seeds. Total DNA was extracted from each spiked homogenate as described by Ioos *et al*.^24^ using the Nucleospin Plant II miniprep (Macherey-Nagel) DNA extraction kit. For each level of contamination, all the DNA extracts were pooled, homogenized, and then distributed as 50 µL *F*. *circinatum* contaminated seed DNA to be used as template for PCR testing (F85 to F92, Table 5). In total, each laboratory received eight pine seed DNA extracts anonymously labeled, which were transported using a fast delivery service and kept in a freezer until analysis.

In total, each partner received an identical panel of 79 DNA extracts, to be tested in duplicate analysis for each protocol that was assessed.

### Data generation and analysis

#### Indeterminate results

Data were processed anonymously, and no communication was allowed about the trials between partners before the end of the collaborative study. For each protocol, each participant tested all 79 DNA extracts in duplicate. For each sample, results of the tests were reported as either “positive” or “negative”, based on the duplicate analyses. With the exception of protocol p8 (where a Ct < 36 should be considered as a positive result), none of the five published real-time PCR protocols recommended a decision cut-off value. Therefore, the decision to rate a DNA sample as “positive” or “negative” was up to each participant, following the decision rules in force in the laboratory. However, in case of doubt or difficulty in interpretation of the results, “indeterminate result” could be reported. The participants were nevertheless encouraged to submit a brief description of the problem encountered. Indeterminate results between protocols, as reported by the participating laboratories, were compared by Fisher’s exact tests for count data.

Indeterminate results were also compared between laboratories by protocol using Fisher’s exact tests. Ideally, these comparisons would have been performed comparing all indeterminate results generated by laboratories, across all tests. However, it must be noted that not all participants implemented all nine protocols, and missing data for some partners exist in the datasets (for example laboratories that only participated in one test would not be included in the statistical test). Therefore, the decision was made to compare laboratories by protocol, in order to have an idea of potential differences that can exist for example between equipment, location or staff in charge of the tests. In both cases, Fisher’s exact tests were performed with simulated P-values based on 1 × 10^5^ replicates.

Indeterminate results were then transformed following two scenarios as suggested by Chabirand *et al*.^47^ and Loreti, *et al*.^48^. The first scenario considered that an indeterminate result would be further assessed by the laboratory, and would always be rated “as expected” (i.e. a sample containing *F*. *circinatum* DNA would be rated as positive, and a sample not containing *F*. *circinatum* DNA would be rated as negative), so that the participant would always make the right decision, eventually. This dataset is here referred to as Dataset 1 (DS1). In the second scenario, an indeterminate result would always be rated “not as expected” (i.e. a sample containing *F*. *circinatum* DNA would be rated as negative, and a sample not containing *F*. *circinatum* DNA would be rated as positive), so that the participant would always make the wrong decision. This dataset is here referred to as Dataset 2 (DS2).

As the objective was to show the potential biases in the application of a protocol that may arise through differences between equipment, location or staff in charge of the analysis, identity of laboratories is not revealed and only the range of indeterminate results is shown.

#### Rates of false positive and false negative results

Results of the protocols were assessed by computing a number of parameters using both datasets: (i) PA, the number of positive accords or true positives, defined as the number of DNA samples from *F*. *circinatum* strains (or DNA from seed samples contaminated with *F*. *circinatum*) yielding positive results with the protocol; (ii) NA, the number of negative accords or true negatives, here defined as the number of DNA samples from other *Fusarium* species yielding negative results with the protocol; (iii) PD, the number of positive deviations or false positives, which takes into account the number of DNA samples from other *Fusarium* species (or DNA from seed samples not contaminated with *F*. *circinatum*) yielding positive results with the protocol; and iv) ND, the number of negative deviations or false negatives, which corresponds to the number of DNA samples from *F*. *circinatum* strains (or DNA from seed samples contaminated with *F*. *circinatum*) yielding negative results with the protocol.

Similarly, for both datasets, the performance of the protocols regarding specificity was assessed using the PD rate, computed as PD rate = 100 × (number of misclassified known positive samples/total number of known negative samples), and the ND rate, computed as, ND rate = 100 × (number of misclassified known negative samples/total number of known positive samples). As for indeterminate results, the PD and ND rates were compared between laboratories by protocol (in total nine comparisons) for both datasets.

All comparisons were performed using Fisher’s exact tests for count data with simulated P-values based on 1 × 10^5^ replicates.

#### Other performance criteria

For each protocol and for each participant the results obtained for the blind samples were processed according to EN ISO 16140 standard^49^ and the PM7/98 (2) EPPO standard^16^. Three performance criteria were assessed: relative accuracy (AC), diagnostic specificity (SP) and diagnostic sensitivity (SE). AC represents the agreement between the expected results and the results obtained using the protocol. SE provides an estimation of the ability of the procedure to detect the target when it is present (presence of *F*. *circinatum* DNA). SP provides an estimation of the ability of the procedure not to detect the target when it is not present (no *F. circinatum* DNA present in the sample). AC, SP and SE were estimated using PA, NA, PD and ND, described in the previous sections, as follows:

$$\begin{array}{rcl}{\rm{AC}} & = & 100\ast ({\rm{PA}}+{\rm{NA}})/({\rm{NA}}+{\rm{PA}}+{\rm{PD}}+{\rm{ND}})\\ {\rm{SP}} & = & 100\ast {\rm{NA}}/({\rm{NA}}+{\rm{PD}});\\ {\rm{SP}} & = & 100\ast {\rm{PA}}/({\rm{PA}}+{\rm{ND}});\end{array}$$

Tests on the equality of SE, SP and AC between methods were performed using Fisher’s exact test.

Qualitative reproducibility or concordance (CO) was also estimated for each protocol. Concordance is the probability that two identical test materials sent to different laboratories will both provide the same results (i.e. both found positive or both found negative)^50^. Concordance for qualitative analyses is similar to reproducibility for quantitative analyses, and this performance criterion is a means to assess the ability of a protocol to provide consistent results with identical samples that are tested under different conditions: operator, equipment, master mix or DNA polymerase brand, location, time^49^. In order to have a reliable estimation of CO, it was calculated for each protocol using the original data reported by the participating laboratories. This means that positive, negative and indeterminate results were included. CO between protocols was compared for both datasets using Fisher’s exact tests for count data.

#### Statistical software for data analysis

All statistical tests were performed using the R statistical software version 3.4.0^51^. Statistical tests were considered as significant for estimated P-values with a confidence of less than 5%. All figures were produced using the R package “ggplot2”^52^.

## Supplementary information

Supplementary information

Supplementary dataset 1

## Data Availability

All data generated or analyzed during this study are included in this published article (and its Supplementary Information and Dataset Files).
