# Supplementary material for: Transferability of PCR-based diagnostic protocols: An international collaborative case study assessing protocols targeting the quarantine pine pathogen Fusarium circinatum
Source: Sci Rep. 2019 Jun 3;9:8195. doi: 10.1038/s41598-019-44672-8 (PMC6546748; doi:10.1038/s41598-019-44672-8)
Supplement: Supplementary file 1 — Supplementary information [file 41598_2019_44672_MOESM1_ESM.pdf]

SUPPLEMENTARY INFORMATION

Transferability of PCR-based diagnostic protocols: An international collaborative case study assessing protocols targeting the quarantine pine pathogen *Fusarium circinatum*

Renaud Ioos[1*], Francesco Aloi[2,24], Barbara Piškur[3], Cécile Guinet[1], Martin Mullett[4,22], Mónica Berbegal[5], Helena Bragança[6], Santa Olga Cacciola[2], Funda Oskay[7], Carolina Cornejo[8], Kalev Adamson[9], Clovis Douanla-Meli[10], Audrius Kačergius[11], Pablo Martínez-Álvarez[12], Justyna Anna Nowakowska[13], Nicola Luchi[14], Anna Maria Vettraino[15], Rodrigo Ahumada[16], Matias Pasquali[17], Gerda Fourie[18], Loukas Kanetis[19], Artur Alves[20], Luisa Ghelardini[21], Miloň Dvořák[22], Antonio Sanz-Ros[23], Julio J. Diez[12], Jeyaseelan Baskarathevan[25], and Jaime Aguayo[1].

[1] ANSES Laboratoire de la Santé des Végétaux, Unité de Mycologie, Domaine de Pixérécourt Bât. E, 54220 Malzéville, France.

[2] Department of Agriculture, Food and Environment, University of Catania, Via Santa Sofia, 100, Catania, 95123, Italy

[3] Slovenian Forestry Institute, Department of Forest Protection, Večna pot 2, SI-1000 Ljubljana, Slovenia

[4] Forest Research, Alice Holt Lodge, Farnham, Surrey, GU10 4LH, United Kingdom.

[5] Instituto Agroforestal Mediterráneo, Universitat Politècnica de València, Camino de Vera s/n 46022 Valencia, Spain

[6] Instituto Nacional de Investigação Agrária e Veterinária I.P. (INIAV I.P.), Quinta do Marquês, 2780-159, Oeiras, Portugal

[7] Çankırı Karatekin University, Faculty of Forestry, 18200, Çankırı, Turkey

[8] Swiss Federal Institute for Forest, Snow and Landscape Research WSL, Zuercherstrasse 111, 8903 Birmensdorf, Switzerland

[9] Institute of Forestry and Rural Engineering, Estonian University of Life Sciences, 51006 Tartu, Estonia

[10] Julius Kühn-Institut, Institute for National and International Plant Health, Messeweg 11-12, 38104 Braunschweig, Germany

[11] Lithuanian Research Centre for Agriculture and Forestry, Vokė Branch, Zalioji Sq. 2, 02232 Vilnius, Lithuania

[12] Sustainable Forest Management Research Institute, University of Valladolid – INIA / Department of Vegetal Production and Forest Resources, University of Valladolid, 47011 Palencia, Spain

[13] Cardinal Stefan Wyszynski University in Warsaw, Faculty of Biology and Environmental Sciences, Wóycickiego 1/3 Street, 01-938 Warsaw, Poland

[14] Institute for Sustainable Plant Protection - National Research Council (IPSP-CNR), Via Madonna del Piano 10, I-50019, Sesto Fiorentino, Florence, Italy.

[15] Department for Innovation in Biological, Agro-food and Forest Systems (DIBAF), University of Tuscia,via S. Camillo de Lellis, snc, 01100 Viterbo,Italy

[16] Bioforest S.A. Camino a Coronel km 15 S/N. 4030000 Concepción, Chile.

[17] Department of Food, Environmental and Nutritional Sciences, University of Milan, via Celoria 2, I-20133, Milano, Italy

[18] Department of Biochemistry, Genetics and Microbiology, Forestry and Agricultural Biotechnology Institute (FABI), University of Pretoria, 0028 Hatfield, Pretoria, South Africa

[19] Department of Agricultural Sciences, Biotechnology, and Food Science, Cyprus University of Technology, 3036 Limassol, Cyprus

[20] Departamento de Biologia, CESAM, Universidade de Aveiro, 3810-193 Aveiro, Portugal

[21] Dipartimento di Scienze delle Produzioni Agroalimentari e dell'Ambiente (DISPAA), University of Florence, 50144 Florence, Italy

[22] Phytophthora Research Center, Department of Forest Protection and Wildlife Management, Faculty of Forestry and Wood Technology, Mendel University in Brno, Zemědělská 3, 613 00 Brno, Czech Republic

[23] Forest Health Center of Calabazanos. Regional Government of Castilla y León, JCyL. Poligono Industrial de Villamuriel, S/N, 30190. Villamuriel de Cerrato, Palencia, Spain

[24] Dipartimento di Scienze Agrarie, Alimentari e Forestali, Università degli Studi di Palermo, Viale delle Scienze, 90128 Palermo, Italy

[25] Plant Health & Environment Laboratory, Diagnostic and Surveillance Services, Biosecurity New Zealand, Ministry for Primary Industries, PO Box 2095, Auckland 1140, New Zealand

* Correspondance to renaud.ioos@anses.fr

Supplementary information 1

Protocol 8: Real-time PCR assay parameters and conditions targeting *Fusarium circinatum*

Primers and probe: The primers and probe were designed based on *F. circinatum* SCAR marker sequence (Baskarathevan et al. unpublished)

FC-2F (Forward primer) : 5'-GGATCACATGTAACATAATGTG-3'

FC-3R (Reverse primer) : 5'-TAAGCTCAGACTATCACCT-3'

FC-3P (Probe) : 5'-**FAM**-CGGGATTCTCCCCGATGGTGTTCA-**BHQ1-3'**

PCR reaction (20 µL Volume):

**Reagents**	**Volume per reaction (µL)**
Sterile water	5.0
Reaction Mix (x2)	10.0
5 µM FC-2F (300 nM)	1.2
5 µM FC-3R (300 nM)	1.2
5 µM FC-3P (150 nM)	0.6
DNA template	2.0
Total Volume	20.0

Note: This assay has been tested with PerfeCTa™ qPCR ToughMix™ (Quanta Biosciences) and LightCycler 480 Probes Master (Roche). Any alternative qPCR master mix can be used. However, the test should be validated with relevant positive controls.

PCR cycling conditions

Step	Time	Temp	Cycles
Initial denaturation	5 min	95ºC	Hold
Denaturation	15 sec	95ºC	x 40 cycles
Annealing and Extension	40 sec	62ºC	

Result

A Cq value of <36 is considered as positive detection of *F. circinatum.*

Supplementary information 2

Recommended amplification conditions of each protocol for the detection of *F. circinatum* according to the original publication

**Protocol [a]**	**Forward/ reverse primers**	**Probe**	**Recommended DNA Polymerase / master mix[b]**	**Final concentrations**			**Amplification parameters [/T(°C)/time(S)]**					**Nb Cycles**
				Primers (µM)	Probe (nM)	$MgCl_2$ (mM)	ID[c]	denaturation	Annealing	Extension	Final elongation	
**p1[23]**	10Fcirc-F / 10Fcirc-R 30Fcirc-F / 30Fcirc-R	n.a.	Taq DNA Polymerase (Roche Applied Science)	0.5	n.a.	1.5	94/120	94/30	60/30	72/60	72/420	30
**p2[24]**	FCIR-F / FCIR-R	FCIR-P	qPCR core kit no rox (Eurogentec)	0.3	0.1	5	95/600	95/15	70/55		n.a.	40
**p3[27]**	Fcircinatum_F315 / Fcircinatum_R775	Fcircinatum_T717	Quantitect Multiplex NoRox	0.6	0.1	Unkn.	95/900	95/15	60/90		n.a.	50
**p4[22]**	CIRC1A / CIRC4A	n.a.	Platinum Taq Polymerase (Invitrogen) + SYBR Green	0.5	n.a.	5	94/180	94/35	66/55	72/50	72/720[e]	45
**p5[26]**	CIRC1A / CIRC4A	n.a.	SoFast EvaGreen Supermix	0.1	n.a.	Unkn.	-	95/10	65/15	72/30	- [e]	44
**p6[25]**	CIRC1L / CIRC4L	n.a.	Platinum Taq Polymerase (Invitrogen) + SYBR Green	0.4		Unkn.	95/180	95/35	66/55	72/50	- [e]	45
**p7[28]**	Fcrc-F Fcrc-R	Fcrc-P	TaqMan Universal Master mix (Appl. Biosystems)	0.3	0.2	Unkn.	94/600	95/30	60/60	-	-	50
**p8[SF1]**	FC-2F / FC-3R	FC-3P	Perfecta qPCR ToughMix, quanta biosciences	0.3	0.15	Unkn.	95/300	95/15	62/40	-	-	40
**p9[13]**	CIRC1A / CIRC4A	-	Not specified [d]	0.5	-	2 mM	94/180	94/35	66/55	72/50	72/720	45

[a] Reference
[d] Brand / type recommended in the original paper but study participants were free to use others
[c] Initial denaturation
[d] Not specified in EPPO (2009), but the original work of Schweigkofler *et al.* (2004) reports the use of Platinum Taq Polymerase (Invitrogen)
[e] Melt curve analysis required

Supplementary Information 3

Thermal cycler model and reagent details used by each of the participating laboratories

Laboratory	Reagent PCR	Reagent Sybr-Green PCR	Reagent hydrolysis probe PCR	PCR equipment	Real-time PCR equipment
**Lab1**	n.a.	KAPA BIOSYSTEMS SYBR FAST Universal qPCR kit (Roche)	n.a.	n.a.	QuantStudio 6 Flex System (Applied Biosystems)
**Lab2**	5x HOT FIREPol Blend Master Mix with 7,5 mM MgCl2 (Solis BioDyne)	5x HOT FIREPol Probe qPCR Mix Plus, no rox (Solis BioDyne)	5x HOT FIREPol Probe qPCR Mix Plus, no rox(Solis BioDyne)	T Professional (Biometra)	Rotor-Gene Q (Qiagen)
**Lab3**	Taq DNA Polymerase (Qiagen)	n.a.	qPCR Core Kit No ROX (Eurogentec)	T Professional (Biometra)	StepOnePlus (Applied Biosystems)
**Lab4**	KAPATaq Ready Mix (Roche)	n.a.	n.a.	Techne Prime Thermal Cycler (Cole-Parmer)	n.a.
**Lab5**	Taqmix BioMIX (Biolin)	n.a.	n.a.	MJ Mini thermal cycler (MJ Research)	n.a.
**Lab6**	HotBegan Taq DNA polymerase (Canvax Biotech SL)	Sybr Premix Ex Taq (Takara)	Premix Ex Taq -Probe qPCR (Takara)	Verity Thermal Cycler (Applied Biosystems)	Rotor-Gene Q (Qiagen)
**Lab7**	HGS Diamond Taq (Eurogentec)	Mesa green master mix (Eurogentec)	Core kit no ROX (Eurogentec)	GeneAmp 9700 (Applied Biosystems)	Rotor-Gene Q (Qiagen)

Laboratory	Reagent PCR	Reagent Sybr-Green PCR	Reagent hydrolysis probe PCR	PCR equipment	Real-time PCR equipment
**Lab8**	n.a.	n.a.	Lumino Ct qPCR Ready Mix (Sigma-Aldrich)	n.a.	7500 Real Time PCR System (Applied Biosciences)
**Lab9**	n.a.	n.a.	TaqMan Universal PCR Master Mix (Applied Biosystems)	n.a.	ABI 7500 Fast Realtime (Applied Biosystems)
**Lab10**	Platinum qPCR SuperMix-UDG (Invitrogen)	Platinum SYBRGreen qPCR supermix (Invitrogen)	Maxima Probe/ROX qPCR Master Mix 2X (Thermofisher Scientific)	Rotor-Gene Q (Qiagen)	Rotor-Gene Q (Qiagen)
**Lab11**	n.a.	POWER SYBR GREEN PCR MASTER MIX (Applied Biosystems)	Taq-Man Universal MasterMix (Applied Biosystem)	n.a.	StepOne Plus Real-Time PCR System (Applied Biosystems)
**Lab12**	n.a.	Lightcycler 480 SYBR Green I Master (Roche)	Lightcycler 480 Probes Master (Roche)	n.a.	Lightcycler 480 II (Roche)
**Lab13**	n.a.	Evagreen qPCR Master Mix (Biotium)	n.a.	n.a.	Step one plus (Applied Biosystems)
**Lab14**	n.a.	Power sybr green mastermix (Applied Byosystem)	n.a.	n.a.	Quantstudio 3 (Thermo)

Laboratory	Reagent PCR	Reagent Sybr-Green PCR	Reagent hydrolysis probe PCR	PCR equipment	Real-time PCR equipment
**Lab15**	Taq DNA Polymerase (Roche)	SenseFast SYBR mix (Bioline)	n.a.	Mastercycler nexus gradient (Eppendorf)	CFX96 (Bio-Rad)
**Lab16**	n.a.	KAPA SYBR qPCR Kit (Roche)	KAPA PROBE FAST qPCT Kit (Roche)	n.a.	iQ5 (Bio-rad)
**Lab17**	n.a.	n.a.	PerfeCTa qPCR ToughMix (Quanta Biosciences)	n.a.	Bio-Rad, CFX96 Touch™ (Bio-Rad)
**Lab18**	n.a.	n.a.	QuantiNova Probe PCR Master Mix (Qiagen)	n.a.	CFX96 (Bio-Rad)
**Lab19**	n.a.	n.a.	Perfecta QPCR Toughmix, ROX (Quanta Biosciences)	n.a.	QuantStudio 6 Flex (Applied Biosystems)
**Lab20**	TrueStart Hot Start Taq DNA Polymerase (Thermo Scientific)	n.a.	n.a.	Mastercycler Nexus GSX1 (Eppendorf)	n.a.
**Lab21**	VWR Taq DNA Polymerase (VWR)	n.a.	n.a.	Hybaid PX2 thermal cycler (Thermo Scientific)	n.a.

Laboratory	Reagent PCR	Reagent Sybr-Green PCR	Reagent hydrolysis probe PCR	PCR equipment	Real-time PCR equipment
**Lab22**	Taq DNA Polymerase with Standard Taq (Mg-free) Buffer (New England Biolabs)	n.a.	n.a.	Mj Mini (Bio-rad)	n.a.
**Lab23**	n.a.	n.a.	TaqMan universal Master Mix (Applied Biosystems)	n.a.	iCycler iQ (Bio-rad)

n.a: not applicable
